# Diagnostic accuracy of serological rk-39 test for visceral Leishmaniasis: Systematic review and meta-analysis

**DOI:** 10.1371/journal.pntd.0011938

**Published:** 2024-03-06

**Authors:** Mihret Getnet, Addisu Minaye Dejen, Dessie Abebaw, Getachew Gedefaw Fentahun, Emebet Birhanu

**Affiliations:** 1 Department of Human Physiology, School of Medicine, College of Medicine and Health Sciences, University of Gondar, Gondar, Ethiopia; 2 Department of Epidemiology and biostatistics, Institute of public Health, College of Medicine and Health Sciences, University of Gondar, Gondar, Ethiopia; 3 Department of Pathology, School of Medicine, College of Medicine and Health Sciences, University of Gondar, Gondar, Ethiopia; Instituto de Ciências Biológicas, Universidade Federal de Minas Gerais, BRAZIL

## Abstract

**Background:**

Visceral leishmaniasis (VL), or kala-azar, is a vector-borne tropical disease caused by a group of intracellular hemoflagellate protozoa belonging to the family of Trypanosomatide and the genus *Leishmania*. The disease is distributed around the world and transmitted via the bite of infected female *Phlebotomine* sandflies, and there is variation in the diagnostic accuracy. Therefore, this systematic review and meta-analysis aimed to determine the pooled global sensitivity and specificity of the rk-39 test and to evaluate if there is a difference between the different parts of the world.

**Methods:**

A systematic review and meta-analysis have been conducted on the diagnostic accuracy of dermoscopy. After setting eligibility criteria, literature was searched in four databases and one searching engine. Articles were screened, critically appraised, and extracted independently by two reviewers, and any disagreements were resolved with the involvement of a third person. The quality of the included studies had been assessed by the Quality Assessment of Diagnostic Accuracy Studies (QUADAS 2) tool. Pooled sensitivity and specificity were determined by bivariate random effect analysis. Heterogeneity was assessed by Higgins’s *I*^2^, and when it was present, mitigation was conducted by using sensitivity analysis.

**Result:**

A total of 409 studies were identified, and finally 18 articles were eligible for the review with a total sample size of 5, 253. The bivariate random effect meta-analysis of the 7 diagnostic accuracy studies showed a pooled sensitivity of 0.89 (0.76–0.95) and specificity of 0.86 (0.72–0.94). The +LR was 6.32 (95% CI: 2.85–14.02), the–LR was 0.13 (95% CI: 0.06–0.30), and the diagnostic odds ratio (DOR) was 47.8 (95% CI: 11.3–203.2). Abdel-Latif (2018) was both an outlier and influential for sensitivity, and Walter (2011) was both an outlier and influential for specificity, and removing them from sensitivity and specificity, respectively, was beneficial for reducing the heterogeneity.

**Conclusion:**

Rk-39 is found to have highly accurate measures in the diagnosis of visceral leishmaniasis. Both sensitivity and specificity were found to be highly accurate in the diagnosis of leishmaniasis, with a pooled sensitivity of 0.91 (0.88–0.93) and a pooled specificity of 0.89 (0.85–0.91).

**Ethical consideration:**

As we will use secondary data for the systematic review and meta-analysis, ethical concerns are not necessary.

## Author summary

Leishmaniasis is a vector-borne tropical disease distributed around the world and transmitted via the bite of infected sandflies. There are 3 main forms of the disease: visceral leishmaniasis, which is fatal if left untreated in over 95% of cases and is characterized by irregular bouts of fever, weight loss, enlargement of the spleen and liver, and anemia. The other form of the disease is cutaneous leishmaniasis, which causes skin lesions and life-long scars on exposed parts of the body. Whereas the third one, mucocutaneous leishmaniasis, leads to partial or total destruction of the mucous membranes of the nose, mouth and throat.

In this study, the authors examined the distribution of visceral leishmaniasis, which has significant impact on livelihoods such as agriculture and livestock and affects cognitive, developmental and educational outcomes. Moreover, since visceral leishmaniasis is a killer disease, timely and accurate diagnosis is important to give appropriate treatment. This systematic review and meta-analysis identify the pooled sensitivity and specificity of the rk-39 test regarding its accuracy by considering many studies in the different parts of the world. According to our findings, rk-39 is regarded as a crucial quick diagnostic tool for the diagnosis of visceral leishmaniasis.

## Introduction

Visceral leishmaniasis (VL), or kala-azar, is a vector-borne tropical disease caused by a group of intracellular hemoflagellate protozoa belonging to the family of Trypanosomatide and the genus *Leishmania* [1]. The disease is distributed around the world and transmitted via the bite of infected female *Phlebotomine* sandflies [2,3]. The prevalence is higher in poor populations regardless of a country’s income status and infects more than one billion people in over 140 countries, with 90% of the global VL burden in Africa [4]. Even though, the infection does not contribute significantly to global deaths, it is debilitating and remains the most common infection among the poor worldwide, preventing them from escaping poverty by impacting livelihoods such as agriculture and livestock and affecting cognitive, developmental and educational outcomes [4,5]. The more recent 2017 Global Burden of Disease study estimated Neglected Tropical Diseases were responsible for 62 million disability adjusted life years (DALYs), with 774,000 DALYs from leishmaniasis, and VL contributed 97% of the total DALYs for leishmaniasis, ranking it as the second leading cause of parasitic deaths after malaria [6,7]. It is estimated that about 43.7% of the VL patients are also malnourished [8] and co-infected with HIV [9], and 19% have concomitant malaria [10].

### Diagnostic tests (index and reference tests)

As VL is a killer disease, timely and accurate diagnosis is important to install appropriate treatment [11]. The diagnosis is based on the combination of clinical signs and symptoms with laboratory confirmation [12]. The laboratory analysis is performed by demonstrating *Leishmania* parasites in microscopic preparations taken from the spleen, bone marrow, or lymph node aspirates, which is considered to be the gold standard test [13]. However, the low sensitivity combined with the invasive and risky sample collection procedures deterred the implementation of microscopy in remote endemic areas [14]. To get around the drawbacks of direct parasitological methods, serology has now been put in place in many parts of the globe for the diagnosis of VL [15]. The direct agglutination test (DAT) is launched as the first serological test based on the agglutination of a *Leishmania* promastigote antigen preparation with specific antibodies in patient serum, whose result can be interpreted without any reading aid [16]. The DAT is powerful as a freeze-dried antigen with proven high sensitivity and specificity in all VL-endemic regions around the world with minimal cost [17]. The limitation of DAT is its relatively long overnight incubation, and considering rapid diagnostic tests (RDTs) as alternatives. Especially, Rk-39 RDT detects antibodies against the 39-amino acid repeat antigens encoded by a kinesin-related gene of *Leishmania infantum* [18], is considered to be a good alternative. The rK-39 RDTs are simple to perform, cost-effective, stable at room temperature, and rapid [19]. These immunochromatographic tests are currently widely implemented for the diagnosis of VL in resource-limited developing countries [19–21]. But, here are some drawbacks of rk-39 RDTs: like variable specificity, inability to differentiate between current and past infections, and not being suitable for treatment effectiveness monitoring [22].

Studies performed in many countries around the world that evaluated the diagnostic accuracy of rk-39 RDT and ELISA, showed a large variation with sensitivities, with a range of 27.8% [23] to 100% [18]. Similarly, the specificities also showed a huge variation, from 27.8% [23] to 100% [24]. Despite these variations, there is no worldwide data that shows the diagnostic accuracy of the rk-39 test using RDT or ELISA. Therefore, this systematic review and meta-analysis aimed to determine the pooled global sensitivity and specificity of the rk-39 test and to evaluate if there is a difference between the different parts of the world.

## Methods and materials

### Protocol and registration

A protocol based on preferred reporting items for systematic review and meta-analysis protocols (PRISMA-P) was prepared. It explains all plans for the systematic review and meta-analysis of diagnostic accuracy of rk-39 for visceral leishmaniasis. It has been sent to PROSPERO and registered.

### Eligibility criteria

#### Inclusion criteria

Based on PIRD approach, studies included in the study are: Articles on diagnostic accuracy of rk-39 for visceral Leishmaniasis with all people regardless of their age, gender, country, or race; Studies with Index test of rk-39 performed on serum or blood; Articles which has a known demarcated reference standard to confirm the diagnosis (any reference), The study which provide sufficient data to allow estimation of a marker’s accuracy for a diagnostic marker, the study which directly or indirectly provide at least four values, which are the following: the number of true positives (TP), false positives (FP), true negatives (TN) and false negatives (FN), to (re)construct a two-by-two table, The study which is an original report, the study which assess the ability of one or more markers to detect the presence of visceral leishmaniasis, Those that are written in English language or translated to it despite their date of publication or study design.

#### Exclusion criteria

Studies that involve non-human subjects, duplicates, abstract-only papers(editorials), case reports/series, the unavailability of full text, and systematic reviews. Studies that use human samples other than serum or whole blood were excluded from the study.

### Information sources

Four electronic databases and two search engines were used to search articles. These are: PubMed database on 28 August 2022, Hinari data base on 28 August 2022, Cochrane library on 29 August 2022, Scopus database on 30 August 2022, and Google Scholar searching engine on 29 August 2022 and Google searching engine on 30 August 2022. There was date restriction and we considered studies after 2005.

### Searching strategy

(rk-39) AND ("visceral leishmaniasis" OR "black fever" OR "kala-azar") AND (sero*) AND (diagnos*)

To facilitate the searching process, different searching techniques were used according to the databases and/or searching engines. Phrases were conjoined by quotation to be considered as a single word; truncation was used to involve all alternatives of a specific word; and Boolean operators were used to conjoin keywords, synonyms and/or MeSH words. The searching of the keywords focused on the titles and abstracts, and there was date restriction after 2005.

Finally, by using the above methods, we have gotten a total of 409 articles and added them to the EndNote library. More than half of them were obtained from the database PubMed (249) (Table 1).

**Table 1:** Shows searching strategies for diagnostic accuracy test of visceral leishmaniasis.

PubMed data base				
MeSH Head	Entry Term	Combination of key words	Finding results	Date of searching
Visceral leishmaniasis	Kala-azar Black fever	((("rk-39"[Title/Abstract])) AND ("visceral leishmaniasis"[Title/Abstract] OR "Black Fever"[Title/Abstract] OR "Kala-Azar"[Title/Abstract])) AND (sero*[[Title/Abstract]) AND (diagnos*[[Title/Abstract])	27 articles	August 28,2022
Hinari database				
Visceral Leishmaniasis	Kala-azar Black fever	((TitleCombined:(rk-39)) AND ((TitleCombined: (visceral leishmaniasis)) OR (TitleCombined: (Black Fever)) OR (TitleCombined: (Kala-Azar))) AND (TitleCombined:(sero*)) AND (TitleCombined:(diagnos*))	33	August 28,2022
Scopus database				
Visceral leishmaniasis	Kala-azar Black fever	"Rk-39" AND "visceral leishmaniasis" OR "black fever" OR "kala-azar" AND sero* AND diagnos*	19	August 30,2022
Cochrane database				
Visceral Leishmaniasis	Kala-azar Black fever	(rk-39):ti,ab,kw AND ("visceral leishmaniases"):ti,ab,kw OR ("black fever"):ti,ab,kw OR ("kala-azar"):ti,ab,kw AND ("serological test"):ti,ab,kw	1	August 29,2022
Google Scholar search engine				
Visceral Leishmaniasis	Kala-azar Black fever	diagnose OR diagnosis OR diagnostic OR diagnosing AND serological diagnose AND "kala-azar" OR "black fever" OR "visceral leishmaniasis" AND "rk -39"	249	August 29,2022
Google				
Visceral Leishmaniasis	Kala-azar Black fever	Diagnostic accuracy of serological rk-39 test for visceral leishmaniasis	80	August 30,2022

### Study selection

After exporting all the results of the search to EndNote, two authors (AMD and MG) independently checked the articles, and the first 61 duplicate papers were removed. Then, duplication-free articles were screened further. The title was assessed according to the eligibility criteria, and about 288 unrelated articles were removed. The next task was assessing articles based on their abstract, and we managed to remove 24 of them that were different from the research question. Five articles that have only an abstract were also removed as the full text could not be obtained. Based on our exclusion criteria, one article that was written in Portuguese but had not been translated to English was removed. Three articles which were inaccessible were also removed as they were no-where to be found.

**Fig 1:**
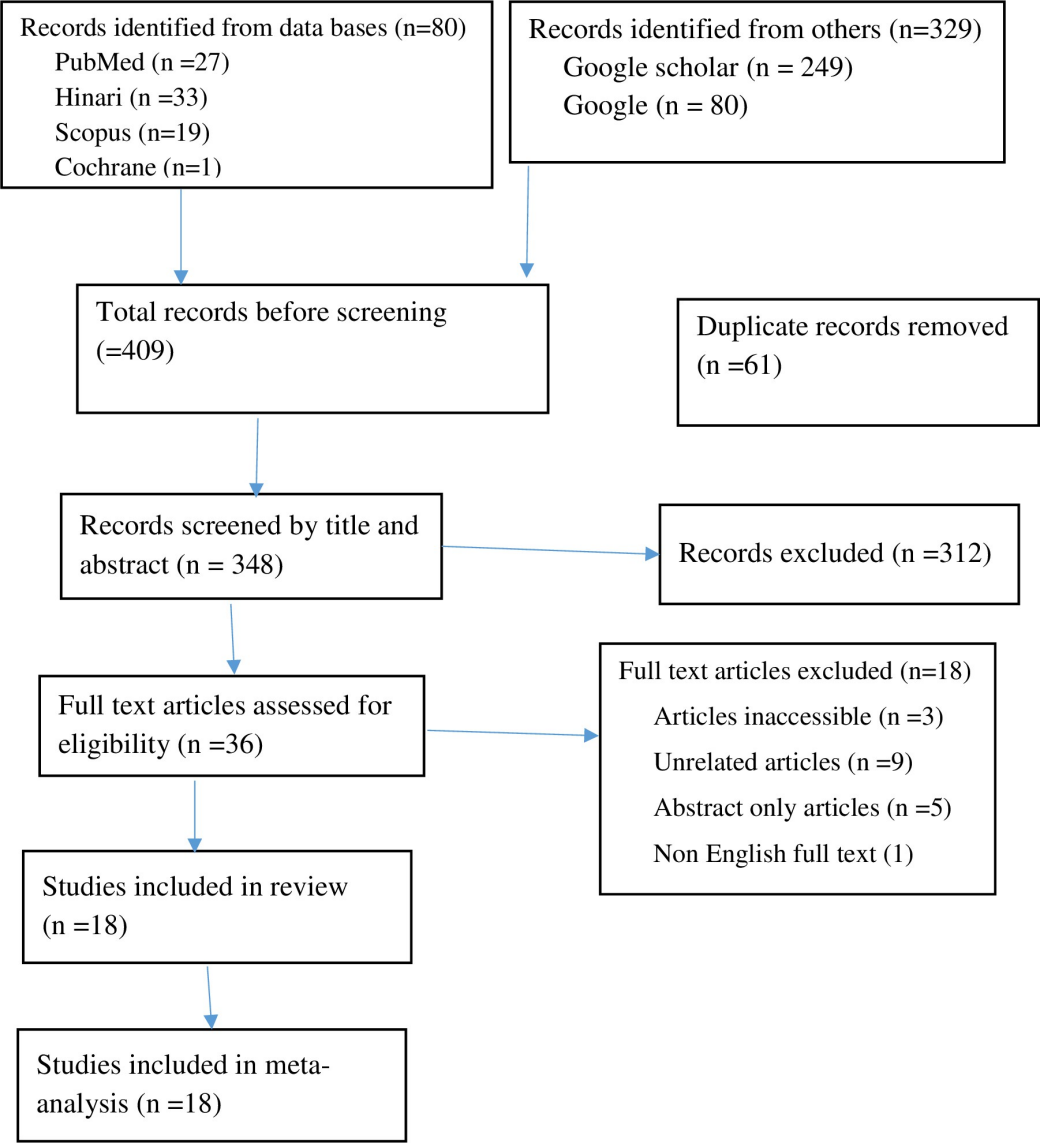
PRISMA flow diagram showing the mechanism of extracting related articles.

Next, full texts that were free to access were downloaded, and the remaining were obtained from different research websites. Then they were evaluated by the two reviewers based on the eligibility criteria. Disagreements between the two reviewers were resolved by a third person involvement in the decision-making. After full-text review, 9 articles were removed due to lack of similarity with the research question. Finally, 18 articles remained after passing all the screening processes, considering they fulfill the inclusion criteria and are appropriate to be added to the systematic review to conduct a meta-analysis. All processes of study selection are illustrated below using the PRISMA flow diagram (Fig 1).

### Data collection process

The data was extracted from the relevant studies by two authors (AMD and MG) independently using the data extraction format that was developed previously to simplify the data extraction process. To assess the agreement level between the two researchers, a preliminary extraction was conducted with some random studies. The data extracted from the full texts was combined into an Excel sheet.

After the two authors extracted the data, they compared and contrasted the data. The discrepancies were resolved by the involvement of a third author to mitigate the difference. Missing data were queried by contacting the authors through email. But as there was no response, the studies were kept, given that they fulfilled the eligibility criteria.

### Data items

As it is a systematic review and meta-analysis of diagnostic test accuracy, the mnemonic for the research question is PIRD. The ‘people’ in the study are all people with a presumptive diagnosis of visceral leishmaniasis, the ‘index’ is rk-39 (rk-39 RDTs produced by InBios International Inc.), (Seattle, WA, USA) or DiaMed-IT Leish, DiaMed AG, Cressiersur-Morat, Switzerland, DiaMed Bio-RAD France, and Kalazar Detect (InBios International, USA, and onsite Leishmania Ab Rapid Test (CTK Biotech, USA)), the ‘reference test’ is any test to diagnose visceral leishmaniasis and is used as a reference by the articles and the ‘diseases’ under study for which the diagnosis is conducted is Visceral Leishmaniasis.

Information that was collected for each study was:

Study information (authors, years of publication, publication journal (if published)); Study characteristics (study design, country and region); Population characteristics (sample size, age and referral pattern (outpatient, ward)); Patient selection (continuous or random sampling); Information related to the index test (type, specification and blinding information); Information regarding reference tests (type, specification and blinding information); The estimated outcome (true positive, true negative, false positive, false negative); and Test accuracy parameters (sensitivity, specificity, positive predictive value, negative predictive value, positive and negative likelihood, diagnostic odds ratio, confidence interval, p-value, clinical symptoms, frequency). In studies that present only accuracy data like sensitivity and specificity with no provision of the outcomes, a two-by-two table was obtained by calculating TP, TN, FP and FN.

### Risk of bias and applicability

Those relevant papers were critically appraised using the Quality Assessment for Diagnostic Accuracy Studies (QUADAS– 2) tool, which is used to assess the risk of bias in diagnostic accuracy studies. It contains 14 items and four domains to describe bias assessment. These are patient selection, index test, reference standard, flow, and timing [21]. The two authors independently appraised the studies, and the disparity between the two people was mitigated by one additional independent person. Each of the 14 items was rated as “yes”, “no” or “unclear”.

**Fig 2:**
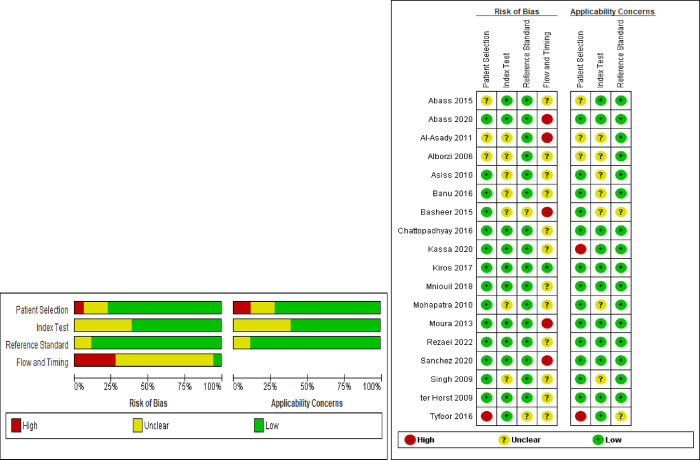
Schematic diagram showing the risk of bias and applicability concern.

By using RevMan version 5.4 software for quality assessment of diagnostic accuracy tests, the risk of bias based on the four QUADAS -2 tool items and applicability concerns for three of the four items were evaluated. These results are presented below (Fig 2).

### Diagnostic accuracy measures

The diagnostic accuracy measures reported on the studies involved in this systematic review and meta-analysis were sensitivity, specificity, positive predictive value, negative predictive value, positive likelihood ratio, negative likelihood ratio and diagnostic odds ratio depending on what the study reported.

### Meta-analysis

Data analysis was conducted by using different softwares in combination namely: Excel, R-software version 4.2.1 (with madad package), Stata version 17 (with metandi, midas and mylabels packages), and Revman version 5.4.

Unlike non-diagnostic meta-analysis, diagnostic meta-analysis is expected to be heterogeneous, which makes the use of a fixed model unnecessary [22]. As the random-effects model gives an estimate of average effect of a DTA by assuming heterogeneity not only due to random variation, but also from natural differences in the magnitudes of test accuracy, it is the recommended model for meta-analysis of DTA studies [25].

In DTA reviews, pooled sensitivity and specificity are summarized according to the bivariate random effect model by considering the correlation between sensitivity and specificity among the included studies [18]. As a summary measures; DOR, LR+, LR-, and AUC were also calculated.

Fagan’s nomogram was used to quantify the post-test probability that a specific individual is affected by visceral leishmaniasis given an observed test result of serological rk-39 and given the probability of the individual having visceral leishmaniasis before the test was conducted (pretest probability) [24].

As the variability of diagnostic accuracy between studies or heterogeneity is the major concern, its presence was tested with *I*^2^ statistics. The value *I*^2^-describes the percentage of the total studies across the primary studies that are more accountable to heterogeneity than chance, and it lies between 0% and 100%. *I*^2^ values of 25%, 50% and 75% are assigned to low, moderate and high heterogeneity, respectively [26]. Also, the HSROC curve was plotted to assess heterogeneity, if the difference between the 95% confidence region and prediction region was larger.

When there was heterogeneity, its causes were assessed in a number of ways. Threshold effect, which is a correlation observed between sensitivity and specificity by varying the threshold for a positive test, was investigated. If a coupled forest plot assumed a V-shape or inverted V-shape, it implies sensitivity and specificity are changing in an inverse way that is suggestive of threshold effect. Additionally, a summary plot was also used to see if there was a curvilinear pattern of individual studies suggestive of a threshold effect. Statistically, Spearman’s correlation was also implied. A Spearman’s correlation coefficient ρ>0.6 shows the presence of a threshold effect [27]. Additional analyses, such as sensitivity and subgroup analysis were implied to determine the cause of heterogeneity.

### Additional analysis

Subgroup analysis was conducted by using the Meta package of R-software to assess source of heterogeneity by classifying studies into groups based on different variables. Three R-packages namely; Meta, Metaphor and Dmetar were used to identify studies that are influential and/or outliers with sensitivity analysis.

Outliers were identified with find.outlier function; when the confidence interval did not overlap with the pooled confidence interval [28]. They were detected by picking up a study with an extremely small or large effect size. After excluding the outliers, the pooled effect without them and the heterogeneity was assessed.

Influential studies were measured using the leave-one-out method with ‘Influence Analysis’ function of R. To use the random effects model, the default of the function (fixed-effect model) was changed to the random-effect model. The pooled results of the meta-analysis were recalculated by leaving out one article each time to identify the influential one. The leave-one-out meta-analysis results sorted by *I*^2^ were used.

Baujat plot was used to recognize influential studies based on their influence on the pooled result of sensitivity and specificity on the vertical axis and their overall contribution to heterogeneity on the horizontal axis [29]. By removing or excluding each individual study, the change on the overall effect of sensitivity, specificity and heterogeneity was assessed.

Publication bias was assessed by using the mylabels package in Stata. But due to the higher false positive results of using Begg’s or Egger’s methods of detecting publication bias by using SE of lnDOR, Deek’s method is preferable for DTA reviews. It plots the lnDOR against 1/effective sample size (ESS) and tests for asymmetry in the plot. A p-value less than 0.10 for the slope coefficient shows the presence of a significant asymmetry, which implies lack of publication bias [30].

## Result

### Study selection

A total of 409 studies were retrieved from PubMed, EMBASE, Hinari, Science Direct, and Google Scholar online databases without the consideration of year or place of publication. After duplicate elimination, 348 articles were screened for the next step.

According to screening based on their titles and abstracts and a careful full-text review of the studies, a total of (n = 43) documents were removed. The 18 remaining manuscripts were included in the systematic review, with a sample size of 5,253.

### Characteristics of included studies

The characteristics of all selected articles assimilated with 26 datasets that used Rk-39 as an index test independently as well as in contrast with other diagnostic tests (like DAT (direct agglutination test), and other different types of rk, such as rk-9, rk-26 and rkLO8). Twelve (33%) of the total studies are from Asia, 11(42.31%) from Africa and three (11.54%) from South America (Table 1). The sample sizes varied between studies from 60 to 1134, and in half of the studies, the sampling was consecutively. The reference standards in most studies (46%) were used only microscopic. All age groups were involved from the minimum age of zero months up to 90 years. Only one (3.85%) study was conducted in a community setting (Table 2).

**Table 2:** Shows the characteristics of selected articles on rk-39 test.

Study	Continent	Sample size	Patient _selection	Age-range	Index Test	Reference test
Abass (2020) [40]	Africa	494	random selection	N/A	rk-39 RDT	Microscopy
Abass etal (2015) [41]	Africa	90	N/A	N/A	rk-39 RDT	Microscopy and culture
Abass etal (2015) [41]	Africa	90	N/A	N/A	rk-39 ELISA	Microscopy and culture
Abass etal (2015) [41]	Asia	66	N/A	N/A	rk-39 RDT	Microscopy and culture
Abass etal (2015) [41]	Asia	66	N/A	N/A	rk-39 ELISA	Microscopy and culture
Al-Asady (2011) [42]	Asia	471	N/A	1month-15years	rk-39 RDT	Microscopy
Alborzi etal (2006) [43]	Asia	184	N/A	3month-5years	rk-39 RDT	Microscopy
Asiss etal (2010) [44]	S. America	332	Consecutive	1 month-76.8years	rk-39 RDT	Microscopy
Banu etal (2016) [45]	Asia	1134	Consecutive	2-75years	rk-39 RDT	Microscopy and culture
Basheer (2015) [46]	Asia	178	Consecutive	8 months to 13 years	rk-39 RDT	Microscopy
Chattopadhyay (2016) [24]	Asia	100	N/A	0-20years	rk-39 RDT	Microscopy
Kassa etal (2020) [20]	Africa	131	random selection	N/A	rk-39 RDT	Microscopy and culture
Kassa etal (2020) [20]	Africa	131	random selection	N/A	rk-39 RDT	Microscopy and culture
Kassa etal (2020) [20]	Africa	131	random selection	N/A	rk-39 RDT	Microscopy and culture
Kassa etal (2020) [20]	Africa	131	random selection	N/A	rk-39 ELISA	Microscopy and culture
Kiros and Regasa (2017) [47]	Africa	62	Consecutive	N/A	rk-39 RDT	Microscopy
Mniouil etal (2018) [48]	Africa	89	Consecutive	6 month- 62years	rk-39 RDT	Microscopy
Mohapatra etal (2010) [49]	Asia	80	Consecutive	N/A	rk-39 ELISA	Microscopy
Moura etal (2013) [50]	S. America	476	Consecutive	0–80 years	rk-39 RDT	IFAT
Rezaei etal (2022) [51]	Asia	355	Consecutive	3 month—16 years	rk-39 RDT	IFAT
Rezaei etal (2022) [51]	Asia	355	Consecutive	3 month—16 years	rk-39 RDT	IFAT
Rezaei etal (2022) [51]	Asia	355	Consecutive	3 month—16 years	rk-39 RDT	IFAT
Sanchez (2020) [52]	S. America	255	random selection	1–90 years	rk-39 RDT	Microscopy and culture
Singh (2009) [53]	Asia	60	Consecutive	0–60 years	rk-39 RDT	Microscopy
Tyfoor (2016) [54]	Africa	175	Consecutive	3–48 years	rk-39 RDT	Microscopy
Ter Horst etal (2009) [55]	Africa	699	Consecutive	All age groups	rk-39 RDT	Microscopy

### Risk of bias and applicability

Generally, the included studies were considered to be of high quality following the most widely accepted Quality Assessment of Diagnostic Accuracy Studies-2 (QUADAS-2) tool and Rev manager software were used to assess the quality of the included studies related to the risk of biases affecting their applicability in 14 items and four domains to describe bias assessment.

The first section was about patient selection, which revealed 75% of the included studies have a low risk of bias respectively, and almost 70.6% of them have no applicability issues. On the other hand, 17.6% of the studies were unclear for both risk of bias and applicability concerns.

The next part is Index test. Risk of bias with respect to the index test was evaluated as unclear in six studies because it was not reported whether the index test (rk-39) was blinded (its test results were interpreted without knowledge) to the test result’s reference standard test. Concerning applicability, all the included studies used rk-39 as their index test, in which its conduct or interpretation flow with the review question.

The reference standard is the third domain, and risk of bias with respect to the reference test ranked unclear in one study: in 94.1% of studies, reference standard test results were interpreted without knowledge of the results of Index test. There was no concern that the reference standard could correctly classify the presence or absence of leishmaniasis. About 5.88% of the studies used references that were unclearly applicable for the interpretation of test results were blinded and reference cut-off value (Fig 2).

The last domain flow and timing showed 23.5% of studies rated as high risk of bias because it was an unclear about an appropriate interval between reference and index tests and/or not all patients were included in the analysis. There was unclear risk of bias in 58.8% of the included studies because it was unclear about the flow or timing of the study (Fig 2).

### Results of individual studies

**Fig 3:**
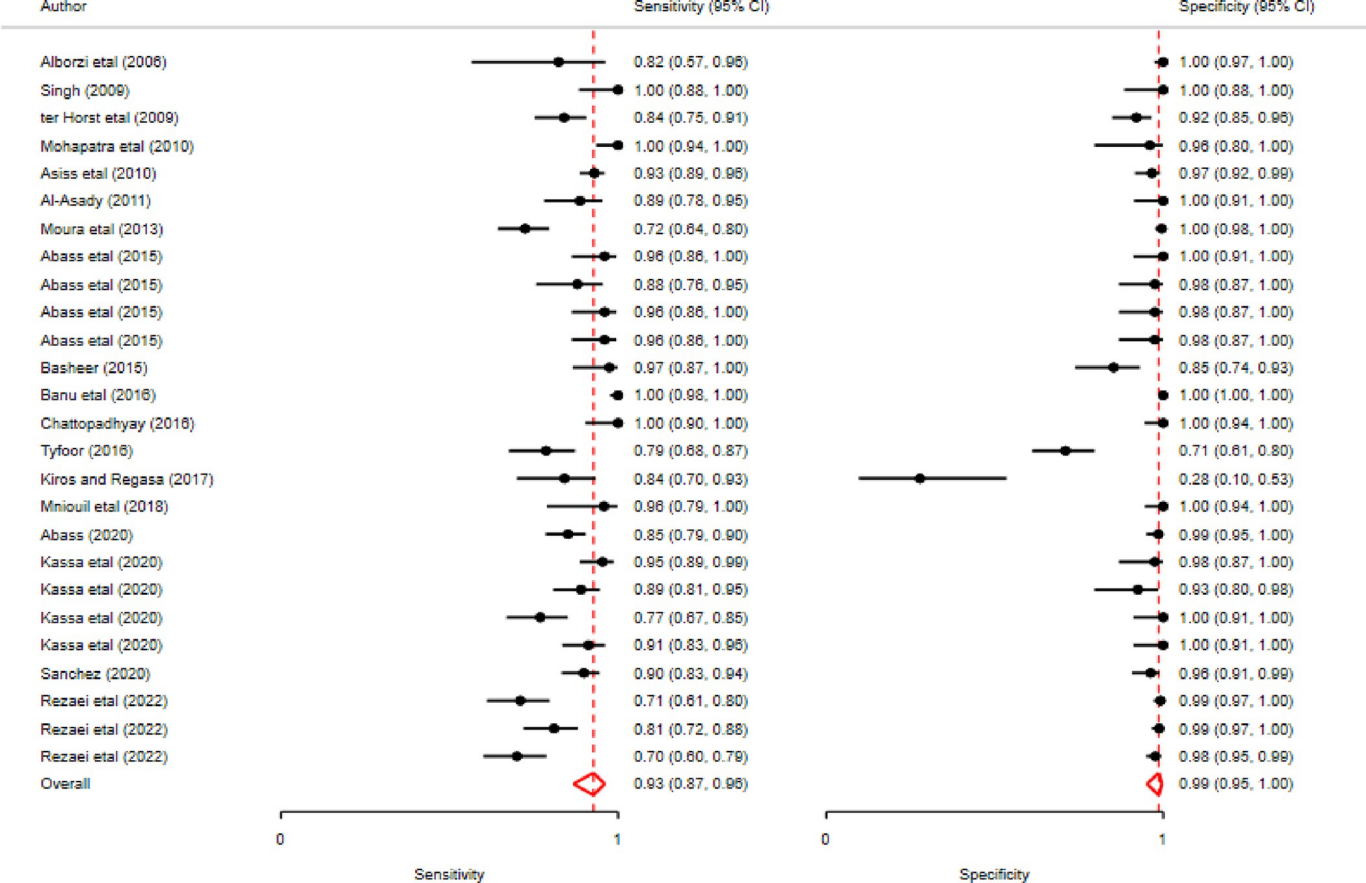
Schematic diagram showing the results of individual studies.

The paired forest plot of individual studies presented the descriptive statistics of each primary study that shows there is considerable variation between studies regarding the sensitivity and specificity of the individual studies (Fig 2). The estimated sensitivity ranges from 0.70 (0.60–0.79) to 1.00 (0.94–1.00) and the specificity varies between 0.28 (0.10–0.53) and 1.00 (0.98–1.00). This relatively high sensitivity and low specificity indicates that most of the studies have few false negative and of high false positive results of disease status (Fig 3).

**Fig 4:**
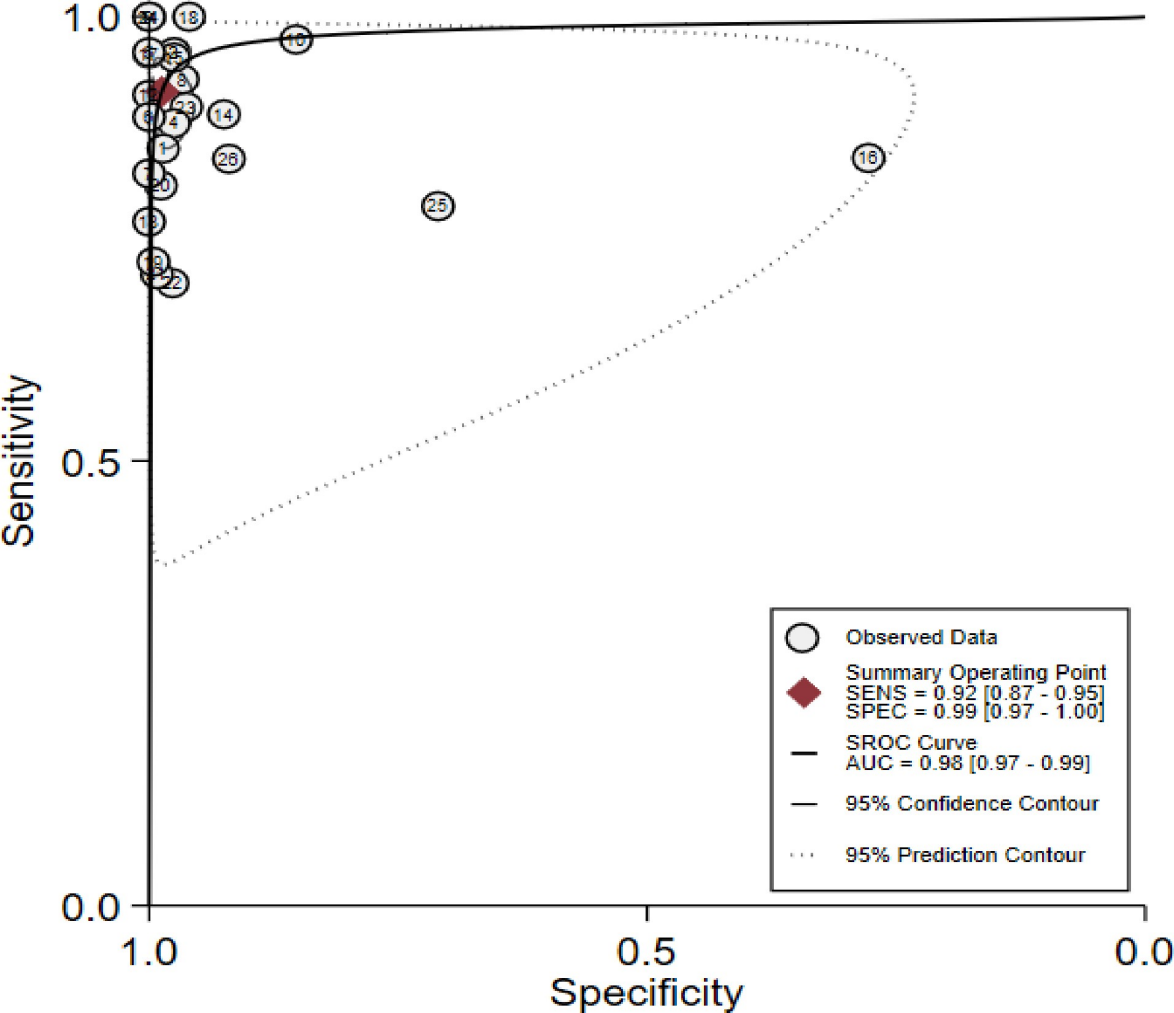
Schematic diagram showing accuracy test.

The meta-analysis revealed that pooled estimates of sensitivity of 0.93 (0.87, 0.95) and specificity 0.99 (0.95, 1.00), as illustrated in Fig 4. According to random effect model estimation, the positive likelihood ratio was around seventy-one (71.22%) with 95% confidence intervals ranging from 27.02 to 187.71, the negative likelihood (–LR) was 0.085 (95% CI 0.05–0.13), and the diagnostic odds ratio (DOR) was 834.1 (95% CI 254.16–2737.59). The area under the ROC (AUROC) curve was 0.98 (0.97, 0.99); this shows high accuracy of the test (Fig 4).

### Fagan nomogram analysis

**Fig 5:**
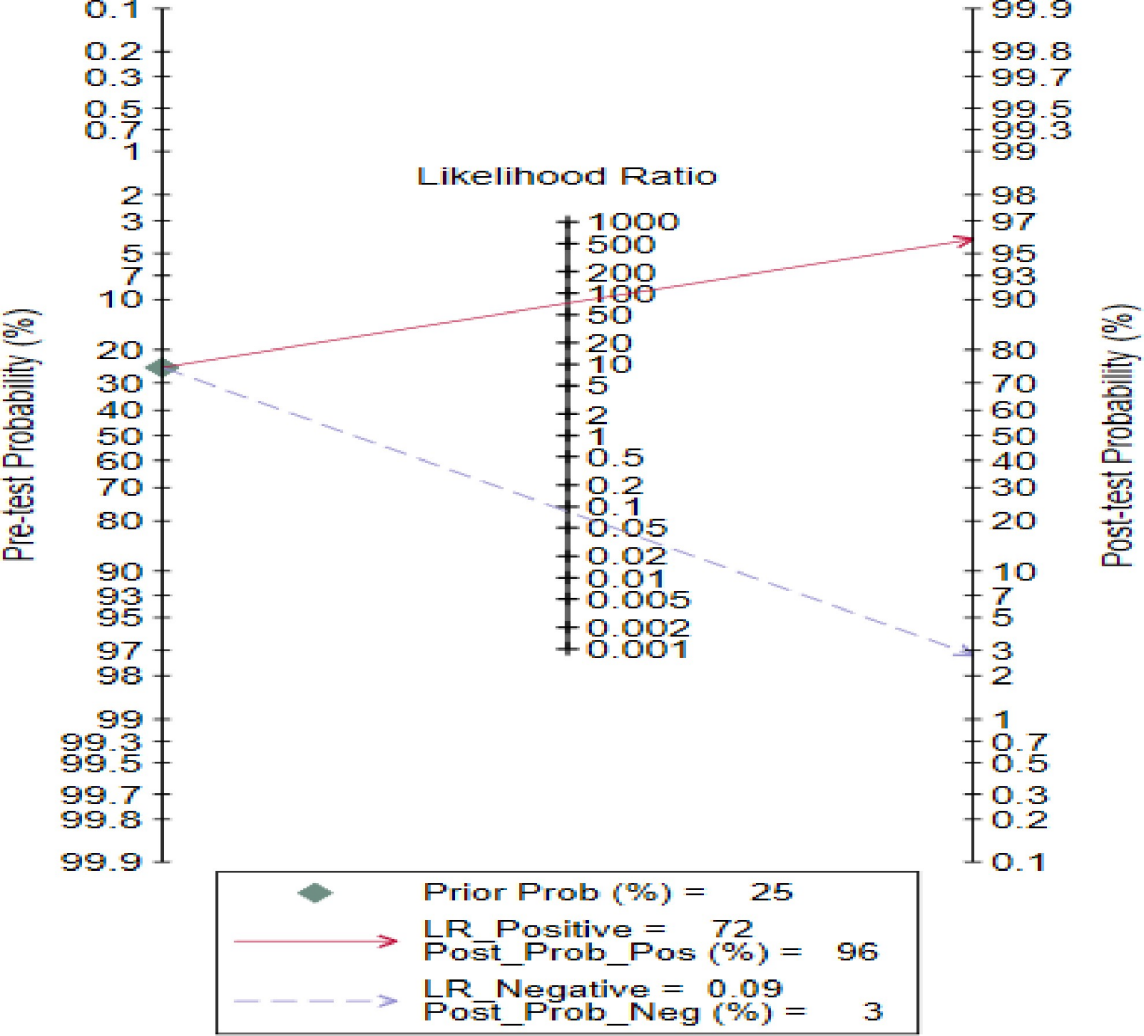
Schematic diagram showing the probability of having leishmaniasis.

A Fagan’s nomogram uses the pretest probability of the outcome and the negative likelihood ratio (LR-) to calculate the posttest probability of diagnosis. The prior probability of Leishmaniasis or the estimated prevalence of Leishmaniasis is 25%, the post-test probability of rk-39 for a single patient to have Leishmaniasis truly indicated positive likelihood ratio (LR+) of 72 is 96%. Moreover, the negative test result produced a negative likelihood ratio (LR-) of 0.09, and the post-test probability for a single patient to truly have Leishmaniasis is 3% (Fig 5).

### Testing of heterogeneity

Heterogeneity between studies is especially common in meta-analyses of diagnostic test accuracy. There are many options to test study-heterogeneity. The heterogeneity statistics between the included studies was shown to be significant (p = 0.00), as indicated by the Chi-square (X^2^) statistic of 127.4 and the I-square (I^2^) statistic of 98 (95% confidence interval (CI) 98–99). The summary HSROC curve is another option that can display only sensitivities and specificities at least as large as the smallest study-specific estimates. The shape of the prediction region is dependent on the assumption of a bivariate normal distribution for the random effects and shouldn’t be overinterpreted; it is intended to give a visual representation of the extent of between-study heterogeneity. The larger difference between the 95% confidence region and prediction region is often considerable.

**Fig 6:**
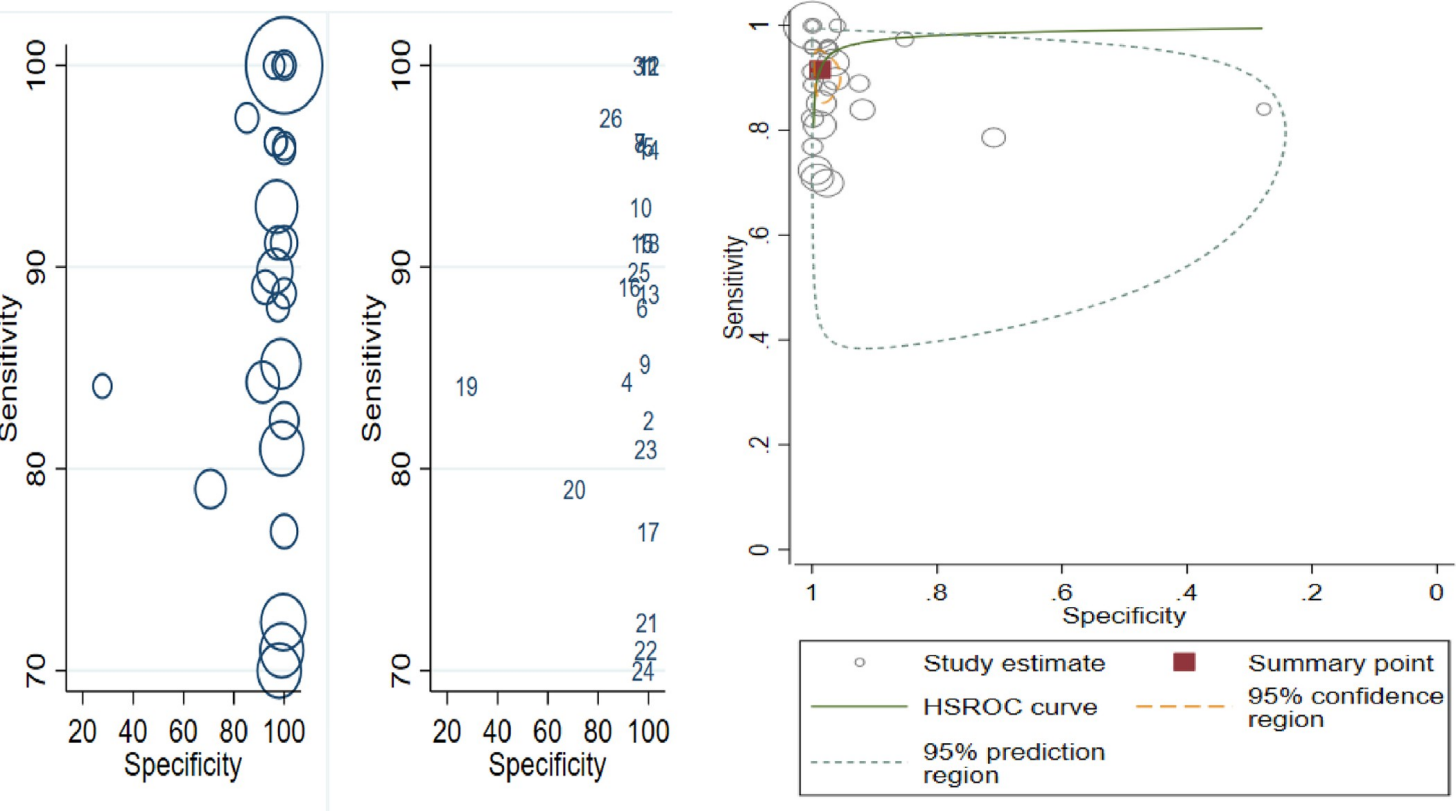
Schematic diagram showing testing heterogeneity among individual studies.

The heterogeneity can be due to differences in study populations. Moreover, if the included studies have variations in their reference standards, heterogeneity due to threshold effect will be under consideration. The proportion of heterogeneity due to threshold effects was 0.13. Spearman’s correlation coefficient between the sensitivity and specificity of all studies can test for the presence of a threshold effect. Spearman’s correlation coefficient r≥0.6 generally indicates a threshold effect. According to our case, Spearman’s rho = 0.0845, p-value = 0.6814, which suggests no threshold effect. Furthermore, a SROC plot can be used to recommend a threshold effect if there is curvilinear distribution of the individual studies in the ROC space that specifies no threshold effect. Therefore, it confirms that the occurrence of heterogeneity was not because of the threshold effect or other sources of heterogeneity using sub-group and sensitivity analysis (Fig 6).

### Empirical Bayes estimates

**Fig 7:**
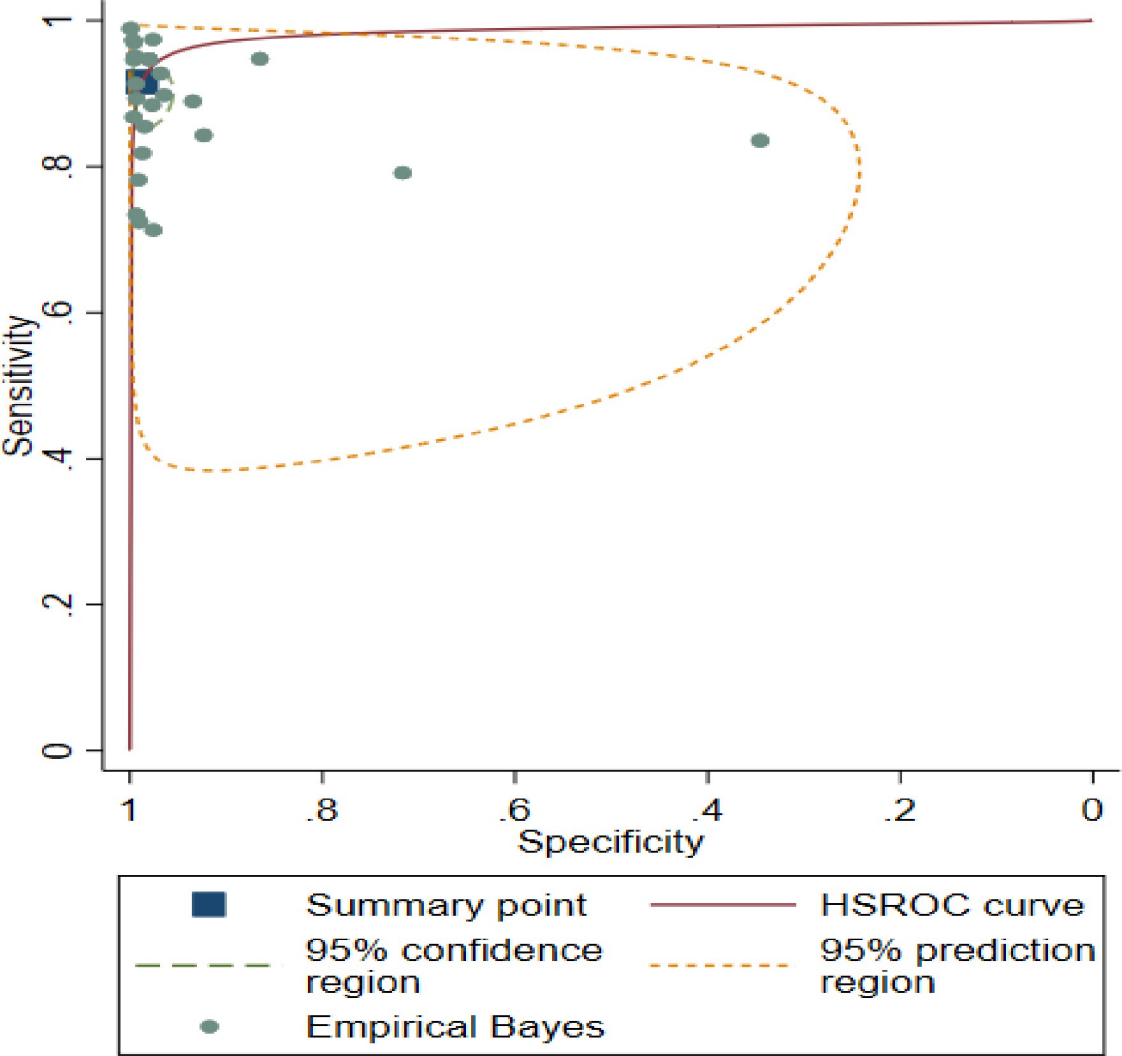
Schematic diagram showing a summary of the best estimates.

Empirical Bayes estimates give the best estimate of the true sensitivity and specificity in each study, and these estimates will be “shrunk” toward the summary point compared with the study-specific estimates shown in Fig 7.

Comparing Fig -6 with Fig -7 shows that the shrinkage is generally greater for sensitivity than for specificity, reflecting both the smaller variance of sensitivity (on the logit scale) and the fact that most studies have fewer participants with disease than without disease, leading to more precise estimates of specificity than of sensitivity.

### Additional analysis

#### Subgroup analysis

The included studies are allocated into subgroups based on: continent (Asia, South America, and Africa); referral pattern (in-patient and community-based settings); patient selection (random and consecutive); reference standard (microscopy, microscopic and culture, IFAT and PCR); index blinding (blinded and non-blinded); and Quality assessment (high, medium and low quality).

The summary sensitivity and specificity of studies in which the interpretation of their index test (rk-39) was done without the knowledge of the reference standard (blinded) is 85.32% (80.04–89.39) and 97.97% (93.77–99.36), respectively. Both sensitivity and specificity were decreased as compared to the bivariate random pooled values. But the heterogeneity still exists significantly (p<0.0001) (Table 3).

**Table 3:** Showing subgroup analysis for selected studies.

Variable	Category	No of studies	Sensitivity			Specificity		
			Pooled (Random effects) (95%_CI)	I^2^	p-value	Pooled (Random effects) (95%_CI)	I^2^	p-value
Continent	Asia	12	96.1% (87.51, 98.86)	64.1%	0.4439	99.27% (97.43, 99.79)	60.5%	<0.0001
	Africa	11	87.92% (83.6, 91.22)	57.2%	0.4439	97.54% (88.37, 99.52)	84.0%	<0.0001
	S. America	3	87.03% (74.93, 93.78)	93.1%	0.4439	98.09% (94.99, 99.29)	51.5%	<0.0001
Referral_ pattern	In_ patient	25	91.89% (87.44, 94.86)	73.7%	0.0281	98.82% (97.05, 99.54)	75.4%	<0.0001
	Community_ based	1	78.67% (67.97, 86.50)	__		71.00% (61.38, 79.04)	__	
Patient_ selection	Random_ selection	6	88.53% (83.44, 92.20)	68.3%	0.0014	97.58% (95.21, 98.90)	90.8%	0.0473
	Consecutive	13	92.36% (82.15, 96.95)	73.4%	0.0014	97.90% (91.73, 99.49)	0.0%	0.0473
	Unknown	7	93.50% (88.74, 96.33)	10.7%	0.0014	99.25% (97.71, 99.76)	0.0%	0.0473
Index blinding	Blinded	15	85.32% (80.04, 89.39)	0.0%	<0.0001	97.97% (93.77, 99.36)	89.9%	<0.0001
	Unknown	11	96.41% (92.13, 98.40)	73.3%	0.0010	99.26% (96.58, 99.84)	11.8%	<0.0001
Type_ Reference Standard	Microscopic	11	92.12% (85.19, 95.96)	29.4%	<0.0001	98.57% (90.00, 99.81)	85.0%	<0.0001
	Microscopic with Culture	10	93.78% (88.62, 96.69)	61.6%	<0.0001	99.08% (96.75, 99.74)	0.0%	<0.0001
	IFAT	4	73.48% (69.19, 77.38)	22.3%	<0.0001	98.82% (97.75, 99.38)	21.6%	<0.0001
	PCR	1	97.44% (83.92, 99.64)	__	<0.0001	85.25% (74.01, 92.14)	__	
Quality_ Assessment	Low_ bias	17	89.26% (83.38, 93.22)	76.9%	<0.0001	98.30% (95.57, 99.36)	81.3%	<0.0001
	Medium_ bias	9	94.47% (87.93, 97.56)	53.5%	<0.0001	99.26% (93.51, 99.92)	62.2%	<0.0001
	High_ bias	0	___	__		___	__	

#### Publication bias

**Fig 8:**
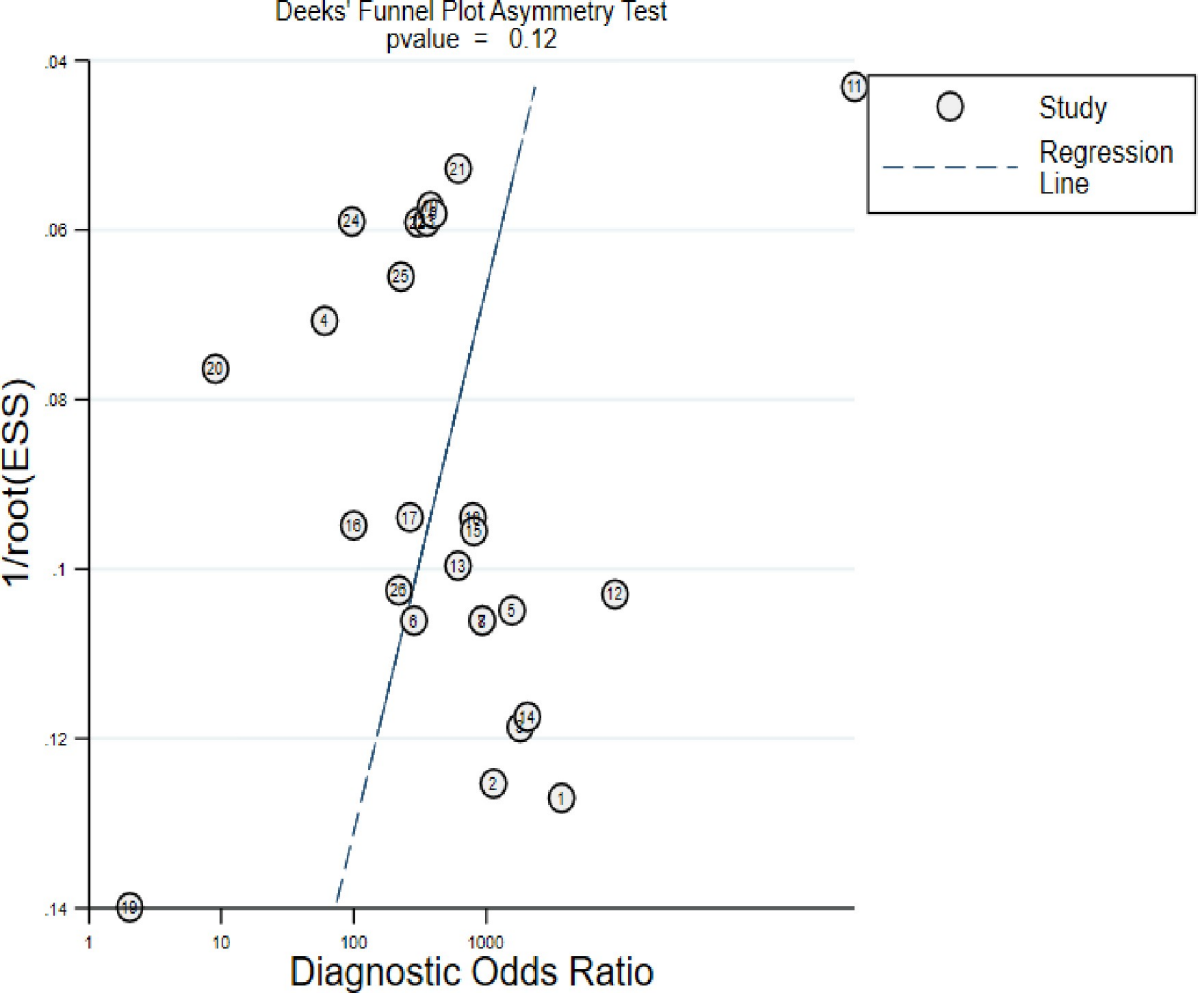
Schematic diagram showing the results of publication bias.

Publication bias is the phenomenon of studies with unfavorable results being less likely to be published than those with more favorable results. If a publication bias occurs, then the published literature is a biased sample of all included studies, and any meta-analysis based on it will be correspondingly biased. The Deek’s funnel plot asymmetry test, which is commonly used to investigate publication and related biases in meta analyses showed that there is no direct evidence of publication bias (P value = 0.117). Since the p-value of the slope is not significant, this can be validated that the datasets in the study are symmetric (Fig 8).

## Discussion

In our systematic review, we noticed that a wide range of sensitivities and specificities of rk-39 RDT among each study (70% to 100% and 28% to 100%) and from the pooled estimates (93% and 99%), respectively. The pooled sensitivity of the rk-39 RDT was in line with a systematic review and meta-analysis (90%) [26] and lower compared to the Asian sub-continent (97%) [27], and it was higher than the pooled sensitivity of the east African studies (85.3%) [27] and Ethiopia (88%) [28]. The possible reasons for this difference may be explained by the commercial brand of rk-39 RDT, the reference test used, and the presence of other comorbidities like HIV, which can lower the performance. Basically, the sensitivity of the kit is different in different parts of the globe. Inspite of being a region with the highest VL burden, sensitivity of the kit in the northern region is lower compared to the other parts of Ethiopia [31]. The essence of the rk-39 RDT is based on the detection of anti-Leishmania antibodies in the patient’s serum, which insist for months or years after a patient is cured of illness. Furthermore, anti-Leishmania antibodies can be produced in asymptomatic and sub-clinical patients as well [29], which may reduce the diagnostic accuracy in high VL burden areas. The other reason may be the genetic diversity of rK-39 gene sequences of *L*. *donovani* strains between sub-continents or perhaps population variation between the continents may be the reason for the discrepancies of performance. Molecular characterization of the rK-39 kinesin repeat sequences of *L*. *donovani* strains from the sub-continents shows a clear divergence, manifested by a difference in drug susceptibility patterns [29].

Unfortunately, there were strain differences across the world [30,32], and the dissemination of certain strains across different geographical areas may cause variation in the effectiveness of the kit [33]. Even though, the northern and north-western Ethiopia strains are similar to those of Sudan [34], whereas the southern strains are similar to the Kenyan strain [35,36], which maybe revealed the reason for performance disparities across the continents. The main challenge of meta-analysis of diagnostic test accuracy is dealing with heterogeneity. In our study, a considerable level of heterogeneity (I^2^ > 75) was detected. To identify the source of heterogeneity, subgroup analysis was done using a different type of reference test; continents, patient selection and quality assessment were carried out. Accordingly, sub-continental variation and quality assessment differences were determined as the main sources of heterogeneity while different types of reference tests variation in patient selection were not. One of the critical parameters to be assessed during meta-analysis, particularly in interventional studies, is publication bias. These techniques, however, are inappropriate for meta-analysis of diagnostic testing [37]. For the purpose of confirmation, we have determined the Deek’s funnel plot asymmetry test p-values and observed a non-significant publication bias, p  =  0.117.

The sensitivity and specificity of rk-39 RDT were higher (93.8% and 99.08%) in studies that used microscopy with culture and/or only microscopy (92.1% and 98.6%) and PCR (97.4% and 85.3%) as reference tests. The possible reason could be that L. donovani complex can cross-react with the test’s specificity through activating B cells in a non-specific way [38], which implies the rk-39 RDT performance differences observed in this analysis.

The diagnostic odds ratio (DOR), which is not influenced by disease prevalence, is an important single quantitative parameter that revealed that the tests could distinguish between individuals with and without diseases [28]. In this meta-analysis, the DOR of rk-39 RDT to diagnose VL was 10.67, and therefore, in comparison to individuals without the disease, VL patients have about 11 times the probability of getting a positive rk-39 test result. The likelihood ratio is another important indicator for the diagnostic test to identify the potential that the VL patients will have a positive test result [39]. In the current meta-analysis, the positive likelihood ratio was 0.96, and hence the positive test result occurs 0.96 times more frequently in VL patients than non-VL patients. Moreover, the negative test result produced a negative likelihood ratio (LR-) of 0.09, and the post-test probability for a single patient to truly have Leishmaniasis is 3%, and hence rk-39 RDT negative test result was 11 times less frequent in VL individuals than the non-VL one. Another important indicator of the performance of a diagnostic test is the HSROC curve, which categorizes affected individuals into VL and non-VL, which is expressed by the AUC. The AUC has different scales: 0.9 to 1.0  =  excellent, 0.8 to 0.9  =  good, 0.7 to 0.8  =  fair, and < 0.5 has no diagnostic value. Therefore, in the meta-analysis, the area under the ROC (AUROC) curve was 0.98 (0.97, 0.99); that shows high accuracy of the test and Spearman’s rho = 0.0845, and hence, according to the result, rk-39 is an excellent alternative diagnostic test for VL.

## Limitations of the study

The primary drawback of this meta-analysis was that each study used a different set of reference tests, which had an impact on the test’s overall sensitivity and specificity. In addition, the rk-39 RDT is based on anti-Leishmania antibodies, which can persist in the serum for a considerable amount of time even after the parasite has disappeared. It has certain inherent limitations, and on top of that, the software did not allow us to execute meta-regression.

This study cannot include gray literature, unpublished manuscripts, and publications before 2005.

## Conclusions

According to our findings, rk-39 is regarded as a crucial quick diagnostic tool for the diagnosis of VL. Aside from the diagnostic accuracy, features like ease of use, speed (10–20 min), low cost, lack of equipment, need for electricity or a cold chain, and reproducibility of results, it is advised to continue using the rk-39 RDT as a diagnostic test, at least in developing countries’ remote Visceral Leishmaniasis endemic areas, until a better tool to test it is developed.

## Supporting information

S1 Data Extracted data to asses diagnostic accuracy of rk-39 serological test. (XLSX)
